# Infected Concha Bullosa with Fungus

**Published:** 2019-03

**Authors:** Hasan-Emre Koçak, Mehmet Keskin, Mehmet-Nurettin Kıral, Hüseyin-Avni Ulusoy, Mehmet Yiğitbay, Kamil-Hakan Kaya

**Affiliations:** 1 *Department of Otorhinolaryngology - Head and Neck Surgery, Bakırköy Dr.Sadi Konuk Training and Research Hospital, Istanbul, Turkey. *

**Keywords:** Bullosa, Concha, Fungus, Infected, Surgery

## Abstract

**Introduction:**

Concha bullosa is the most common variation of the middle turbinate of the paranasal sinuses. When it causes nasal obstruction, osteomeatal unit dysfunction, or rarely chronic infection, surgery is required.

**Case Report:**

We present a fungal infection of concha bullosa, which is a rare indication for surgery of the concha bullosa. A 59-year-old female patient presented with hemifacial pain on the right side, which had lasted for 2 months. There were no pathological findings in her endoscopic nasal examination. Advanced examination by paranasal computed tomography (CT) revealed bilateral concha bullosa variation and soft tissue density in the right concha bullosa. As the biopsy taken from concha bullosa demonstrated fungal hyphae, endoscopic surgical treatment was performed.

**Conclusion:**

We stress the importance of the CT in hemifacial pain by this rare case report, in which endoscopic nasal examination was normal. Fungal infection in the concha bullosa is rare, and infected concha bullosa is a pathology to be considered in the differential diagnosis in patients with complaints of hemicranial headache.

## Introduction

Concha bullosa, the most common anatomical variation of the middle turbinate, which is defined as the pneumatization of it, can be asymptomatic or may cause symptoms such as nasal obstruction, headache, and decrease in the sense of smell. Different studies report the incidence of concha bullosa from 14% to 53.6% (1). In symptomatic patients, partial or total excision of the turbinate can be applied. Although concha bullosa can become infected in some patients, publications on fungal infections of the nose and paranasal region are rare in the literature. However, the number of fungal infections in recent years has risen, which is associated with both an increased life expectancy and the improvements in radiological and endoscopic techniques (2). In this paper, a case of fungal infection of the concha bullosa, which is a rare condition, and which was presented with hemifacial pain on the right, is reported.

## Case Report

**Fig. 1:**
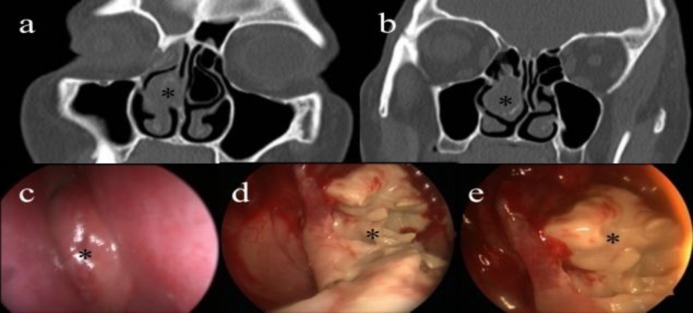
a,b) Paranasal computed tomography image of the infected concha bullosa; c) pre-operative image of the middle turbinate; d,e) View of fungal material in the intra-operative concha bullosa

A 59-year-old female patient, who was monitored at a neurology clinic, was referred to our clinic due to sustained hemifacial pain on the right side. The patient had diagnosed diabetes mellitus type 2, which was under control. There were no pathological findings in the patient's anterior rhinoscopic or endoscopic nasal examination (Fig.1c), although the nasal septum was observed to be deviated to the left. The patient's hematological and biochemical parameters were normal. As an advanced examination, paranasal computed tomography (PNCT) imaging was performed. A radiological examination revealed concha bullosa variation of the bilateral middle turbinates, and soft tissue density in the right concha bullosa (Fig.1a,b). Concha bullosa was incised by applying topical anesthesia to the patient's right nasal cavity, and a punch biopsy was performed. As the pathology report concluded fungal hyphae and necrobiotic material, the patient was prepared for surgery. Upon receiving written consent from the patient, endoscopic sinus surgery under general anesthesia was planned. Lateral and inferior lamellae of the concha bullosa were excised and the infected material inside was submitted to pathologic examination (Fig.1d,e). Upon controlling the bleeding, the intervention was finalized. The patient was prescribed nasal wash and anti-inflammatory treatment post-operatively.

**Fig2:**
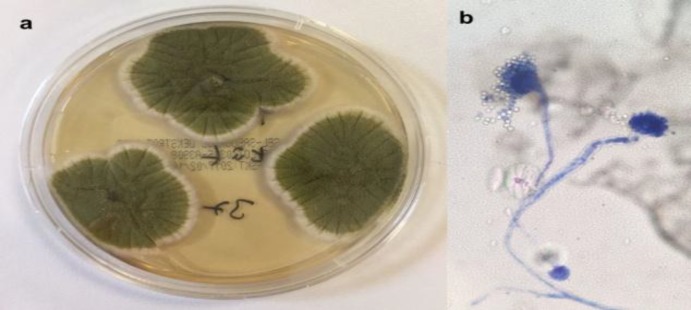
**a.** Microbiological culture image; **b.** Microscobic image

The pathological examination report stated fungal organisms and their hyphae in the necrobiotic material submitted to pathology (Fig.2a,b). "Aspergillus oryzae" was cultured by sequential analysis of the ribosomal DNA genes in the aspirated material submitted for advanced microbiological examination. During the post-operative 6-month follow-up, the patient's headache complaints were diminished and the nasal passage was open.

## Discussion

Concha bullosa, which is characterized by the pneumatization and expansion of the turbinates, is the most common variation of the osteomeatal complex. The condition is encountered in the middle turbinate most frequently, although it can be observed in other turbinates as well. Pneumatization can originate from the frontal recess, agger nasi cells, anterior ethmoidal cells, and middle meatus (3). Concha bullosa can be asymptomatic or can manifest through headache, pain between the eyes, nasal obstruction, or sinusitis due to blockage of the sinus orifice. Although it usually contains air, rarely purulent material, bone septum, pyocele, ossified fibromas, fungal material, and cholesteatoma can be encountered (4). Although the physical and endoscopic nasal examination did not reveal any pathological findings in the presented case, PNCT as an advanced investigation due to the hemicranial pain revealed concha bullosa variation and soft tissue density in the right concha bullosa.

Fungal infections of the nose and paranasal sinuses can cause allergic fungal rhinosinusitis, acute or chronic invasive fungal rhinosinusitis, and fungal balls. The causative fungal agents are most commonly reported as Aspergillus fumigatus, Aspergillus flavus, Scedosporium, Pseudallescheria boydii, and Alternaria. By PNCT imaging, these agents are observed as a mass, which rarely contains calcifications, and which has heterogenous opacity.

In our case presentation, we reported a case with a normal nasal examination, but a diagnosis was made following the PNCT imaging upon suspicion. Bacterial sinusitis, mucocele, pyocele, malignant and benign tumors should be considered in the differential diagnosis. Intraoperatively, the appearance of the fungus is gritty or clay-like. The recurrences are very rare after debridement (5). In our 6-monthly follow ups of the case, no recurrences or complications were encountered.

Aspergillus species have a widespread geographical distribution and are commonly found in nature, particularly in the soil. In our case, we detected Aspergillus oryzae in the cultured material, which is a subspecies rarely reported to cause disease in humans. According to some experts, this species is regarded as a variant of Aspergillus flavus (6). In a review of the literature, we encountered some case presentations with Aspergillus oryzae. Some of these were allergic bronchopulmonary aspergillosis cases in Japan, where it is used in making soy sauce for the Japanese kitchen (7,8). In addition, there were individual case presentations of eye involvement, peritonitis, and meningitis. A review of the literature on the nose and paranasal region revealed a case presentation of a chemotherapy patient as the only published case report on upper respiratory tract in which paranasal sinuses were invaded with Aspergillus oryzae (9).

Despite considerable fungal infection with Aspergillus subtips in concha bullosa in the literature (10-12), to the best of our knowledge, the case we present here is only the second report of upper respiratory tract involvement with Aspergillus oryzae.

## Conclusion

Although fungal infection in the concha bullosa is rare, infected concha bullosa is a pathology to be considered in the differential diagnosis in patients with complaints of hemicranial headache. A paranasal CT may provide input to the differential diagnosis in patients with chronic hemicranial headache and with normal anterior rhinoscopic and endoscopic examination.
